# Successful Clindamycin Therapy of an Infected Subcutaneous Permanent Pacing Lead in a Dog after a Failed Course with Potentiated Amoxicillin and Enrofloxacin

**DOI:** 10.3390/vetsci10020093

**Published:** 2023-01-26

**Authors:** Viktor Szatmári, Astrid M. van Dongen, Mauricio Tobón Restrepo, Marjolein L. den Toom, Niels Jongejan

**Affiliations:** 1 Department of Clinical Sciences, Faculty of Veterinary Medicine, Utrecht University, 3584 CM Utrecht, The Netherlands; 2 Department of Cardiology, Division Heart & Lungs, University Medical Center Utrecht, 3584 CX Utrecht, The Netherlands

**Keywords:** antibiogram, antibiotics, antimicrobial prophylaxis, atrio-ventricular block, beta-lactam, clavulanic acid, lincosamide, pacemaker, ultrasound-guidance, surgical site infection

## Abstract

**Simple Summary:**

An inappropriately slow heart rate can lead to exercise intolerance, collapsing episodes, sudden death and congestive heart failure. Pacemaker implantation is often the only effective therapy to treat these cardiac rhythm disturbances. Unfortunately, pacemaker implantation can have various complications, among others bacterial infection. The best way to clear the infection is to remove the pacemaker, and treat the patient with antibiotics for several weeks before the pacemaker can be re-implanted. Though pacemaker-removal can control the infection, the clinical signs of the slow heart rate will recur and remain uncontrolled. The present case describes a 13.5-year-old dog that got a surgical site infection after pacemaker implantation. Though the first course of antibiotics resolved the clinical signs, the infection recurred. To identify the causative bacteria, the swelling under the skin caused by the bacterial infection was punctured under ultrasound-guidance to gain a sample for laboratory testing. Based on a number of considerations, another antibiotic was chosen, which successfully cleared the infection without the need of pacemaker removal. After completion of this 4-week long antibiotic course, the dog remained free of infection and free from the previously noted clinical signs thanks to the appropriately functioning pacemaker.

**Abstract:**

Though permanent pacemaker implantation is the only effective therapy for certain bradyarrhythmias in dogs, it is not without risks. Bacterial infection of the device is one of the most common complications. Human guidelines recommend besides systemic antibiotics, surgical explantation of the pacing lead and pulse generator in case of device-infection. This report describes a 13.5-year-old dog that received a transvenous endocardial permanent pacemaker because of syncopal episodes resulting from paroxysmal third-degree atrio-ventricular block. Five days after an uneventful surgery, a painful swelling appeared around the subcutaneous part of the lead where this was inserted into the jugular vein. A 4-week course of amoxicillin and clavulanic acid combined with enrofloxacin failed to clear the infection on long-term. Ultrasound-guided puncture of the abscess was performed to gain a sample for bacterial culture and antibiogram. Oral clindamycin of 4 weeks’ duration successfully resolved the infection with *Staphylococcus aureus* without having to explant the device. Repeated ultrasonographic examinations and fine-needle aspiration biopsies were used to evaluate for persistent local inflammation, guiding the length of the antibiotic therapy. Though the described approach has traditionally been ill-advised because of the risk of introducing bacteria and damaging the pacemaker lead, it was successful in our case.

## 1. Introduction

Implantation of a permanent pacemaker is the only effective therapy for a number of bradyarrhythmias in dogs [1,2,3,4,5,6,7]. The most common indications for pacemaker therapy in dogs are third-degree atrio-ventricular block, persistent atrial standstill, sick sinus syndrome, and high grade second-degree atrio-ventricular block that is non-responsive to atropine [1,2,3,4,5,6,7]. 

In dogs, typically a single-lead transvenous endocardial system is implanted, where a single ventricular lead is introduced through the right jugular vein with its tip secured in the right ventricular apex with either passive or active fixation [1,2,3,4,5,6,7,8]. From the jugular venous skin incision, the lead is tunneled subcutaneously to a subcutaneous pocket located either in the right dorsal cervical region or on the right dorsal thoracic region, where the lead is connected to the pulse generator [1,2,3,4,5,6,7,8].

Permanent pacemaker implantation is known to have several possible minor and major complications [9,10,11,12]. One of the most common major complications is bacterial infection of the device [1,2,4,6,9,10,11,12,13,14,15,16,17,18,19]. Both the intracardiac and the subcutaneous parts of the system can be infected. Early postoperative infection is most often caused by introducing bacteria from the skin at the time of the surgery, most commonly *Staphylococci* [14,15,16,17,18]. Clinical signs can vary from local painful swelling to life threatening systemic conditions due to sepsis or endocarditis [14,15,16,17,18]. Systemic antimicrobial therapy alone is thought to be insufficient to solve the infection, mostly because biofilm-formation on the device is suspected to prevent antimicrobials and the host’s immune system to reach the bacteria [14,15,16,17,18]. Therefore, surgical explantation of all parts of the device, i.e., both the pacing lead and the pulse generator, is recommended together with long term, typically 4–6 weeks of, systemic antibiotics to clear the infection [14,15,16,17,18]. After the infection is cleared another pacemaker would be implanted.

Veterinary textbooks and human guidelines warn that puncturing a swelling around any subcutaneous parts of an implanted pacemaker might be detrimental because of potentially damaging the lead and introducing bacteria from the skin [1,2,14,15]. On the other hand, identifying the bacteria that are responsible for the localized or systemic infection is essential for a successful antimicrobial therapy, especially after failure of an empirically chosen antimicrobial course [14,15].

The present report describes a dog, where a cervical abscess developed around the subcutaneous part of a pacing lead in the early postoperative period after permanent pacemaker implantation. As a 4-week course of oral amoxicillin with clavulanic acid combined with enrofloxacin failed to clear the infection on long-term, ultrasound-guided puncture of the subcutaneous fluid accumulation was performed to gain a sample for bacterial culture and antibiogram. Oral clindamycin of 4 weeks’ duration cleared the *Staphylococcus aureus* infection without the need of surgical explanation of the device. 

## 2. Case Presentation

A 13.5-year-old female neutered Border collie dog was referred to the authors’ teaching hospital because of acute onset of exercise intolerance for 1.5 weeks’ duration and syncopal episodes with increasing frequency. The dog was unable to walk longer than 5 min, whereas it used to walk for 30–45 min. Initially, the syncopal episodes took place with exercise only, but in a week’s time its frequency increased, and the dog fainted 3–4 times a day. No other abnormalities were noticed in the dog’s general functioning. The referring veterinarian treated the dog with oral pimobendan (0.21 mg/kg q12h) because of a heart murmur assumed to be caused by myxomatous mitral valve degeneration, and oral carprofen (2.1 mg/kg q12h) for a suspected multifocal arthrosis.

At presentation, physical examination revealed a bright, alert and responsive dog with a body condition score of 6 out of 9 and with a body weight of 24.2 kg. The dog was continuously panting without signs of respiratory distress. The sleeping respiratory rate at home recorded by the owner ranged between 18 and 26 breaths per minute. The femoral pulses were strong, regular, symmetric with a frequency of 60 beats per minute. Mucous membranes were pink with a capillary refill time of less than 1 s. Cardiac auscultation revealed a systolic murmur with the point of maximal intensity at the level of the mitral valve with an intensity of 3 out of 6. No other abnormalities were found on physical examination.

**Figure 1:**
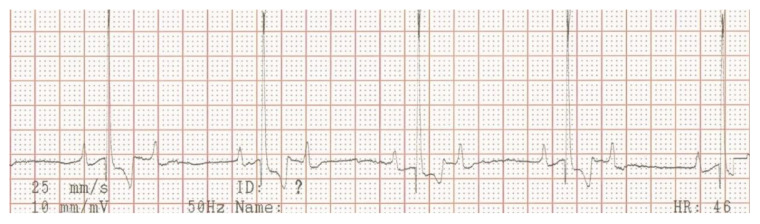
Surface electrocardiogram at initial presentation. Lead aVF shows a second-degree atrio-ventricular block, where every other P-wave is not conducted to the ventricles.

**Figure 2:**
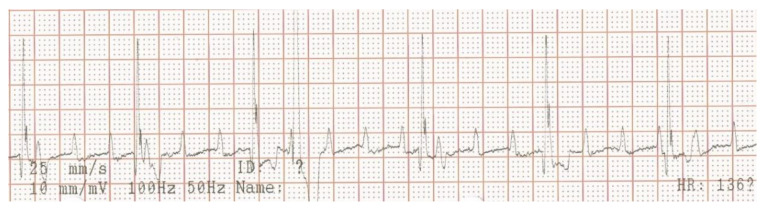
Surface electrocardiogram after intravenous injection of atropine (0.04 mg/kg). Lead aVF shows third-degree atrio-ventricular block with presumed nodal escape beats and a solitary ventricular premature complex. The atrial rate is markedly, and the ventricular rate is mildly increased compared to the pre-atropine situation (Figure 1).

Electrocardiography, echocardiography and blood work were performed as initial diagnostic tests. An ambulatory 6-lead surface electrocardiogram revealed intermittent periods of second-degree atrio-ventricular (AV) block where every other P-wave was unconducted, resulting in a ventricular rate of 46 beats per minute (Figure 1). Intravenously administered atropine-sulphate (0.04 mg/kg) resulted in a third-degree AV-block with a ventricular escape rate of 60 beats per minute, and an atrial rate of 214 beats per minute (Figure 2). 

Echocardiography showed thickened mitral valve leaflets and a moderate mitral valve regurgitation without left atrial or left ventricular dilation, compatible with stage B1 myxomatous mitral valve degeneration according to the ACVIM Consensus Guidelines, 2019. No structural abnormality was noticed at the region of the AV-node. Plasma potassium concentration was within the reference range (3.6 mmol/l, reference: 3.6–5.6 mmol/l). 

**Figure 3:**
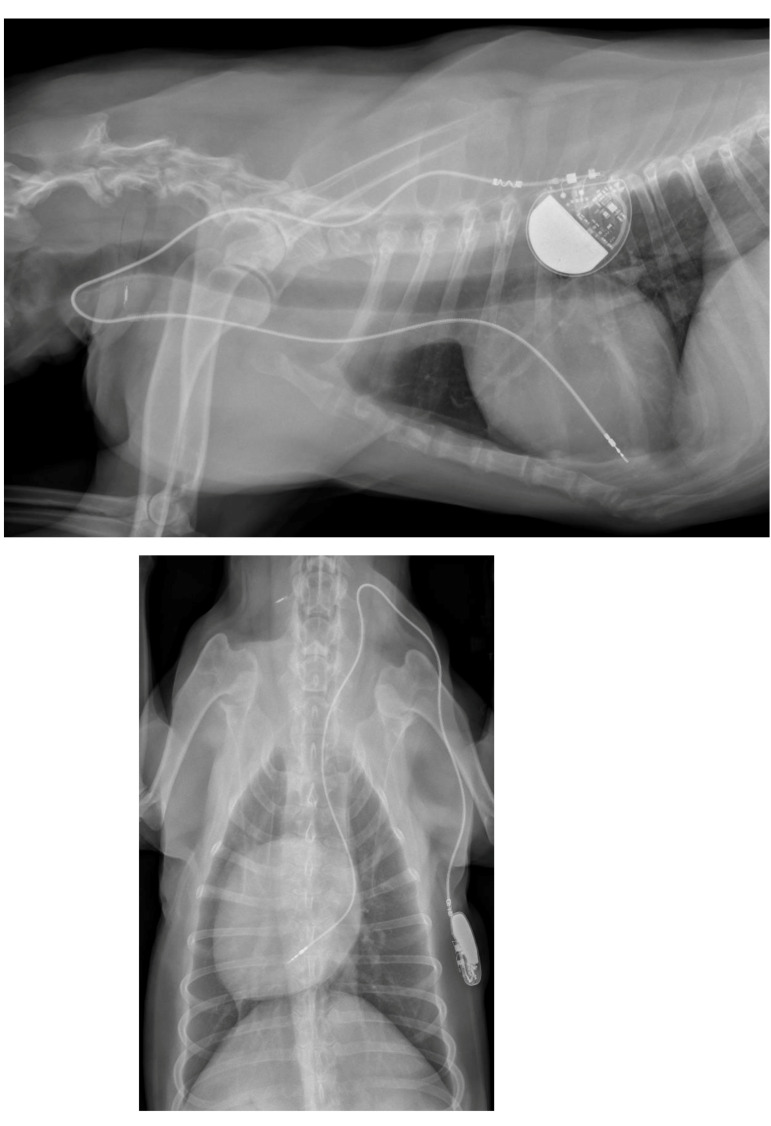
Postoperative thoracic radiographs, lateral and dorso-ventral positions, show appropriate placement of the pacing lead.

A week later, a permanent pacemaker was implanted at the authors’ teaching hospital. General anesthesia was induced with the dog in left lateral recumbency, after placement a temporary pacemaker in the conscious dog via the right saphenous vein into the right ventricular lumen, using a 4 French introducer and a 4 French temporary pacing lead under fluoroscopic guidance. The skin on the right neck and thorax between the chin and the last rib, and between the dorsal and the ventral midline were clipped, and subsequently washed with an antiseptic shampoo containing 4% chlorhexidine gluconate (Hibiscrub, Mölnlycke Health Care, Gothenburg, Sweden). Afterwards, the washed area was sprayed twice with an antiseptic solution containing 0.5% chlorhexidine gluconate and 70% isopropyl alcohol (Hibisol, Mölnlycke Health Care, Gothenburg, Sweden). A surgical skin incision was made and a transvenous permanent pacing lead with active fixation mechanism (58 cm, Capsurefix MRI Surescan 5086, Medtronic, Minneapolis, MN, USA) was implanted through the right jugular vein into the right ventricular apex under fluoroscopic guidance. A second skin incision was made on the right dorsal thoracic wall to create a pocket for the pulse generator. The free end of the pacing lead was subcutaneously tunneled from the cervical incision and connected to the pulse generator (T20 SR, Vitatron, Maastricht, the Netherlands), which was placed dorsally on the right thoracic wall under the cutaneous trunci muscle. The pacemaker was programmed for ventricular bipolar pacing and bipolar sensing in rate responsive mode (VVIR) with a variable frequency between 80–120 beats/minute with a minimum night frequency of 60 beats per minute. At implantation, the stimulation threshold was 0.25 V at 0.40 ms and the lead impedance was 750 Ohm. The pacing amplitude was set at 2.5 V and 0.40 ms, with a sensitivity of 2.5 mV. The pacing lead and the pulse generator were secured to the underlying tissue using non-absorbable multifilament suture material (Ethibond 2-0). The surgical wounds were closed with single interrupted sutures using monofilament suture material in two layers (Monocryl 3-0 for the subcutis and Ethilon 3-0 for the skin). A moisture vapor permeable spray dressing containing acrylic polymer in solvent and propellant mixture (Opsite, Smith and Nephew, Watford, England) was used to cover the two surgical wounds for preventing post-operative surgical site infection. At completion of the implantation procedure, the temporary pacemaker was switched off and the temporary pacing electrode was removed under fluoroscopic guidance. After removal of the introducer from the right saphenous vein, the puncture site was covered with a pressure bandage for 30 min. Postoperative thoracic radiographs were made to document the position of the pacing lead (Figure 3).

The length of the surgery was 107 minutes and the recovery from the general anesthesia was uneventful. Postoperatively, continuation of the previously administered non-steroidal anti-inflammatory drug, carprofen, as a painkiller was recommended. Pimobendan was stopped. The dog was discharged from the hospital on the day of the surgery.

**Figure 4:**
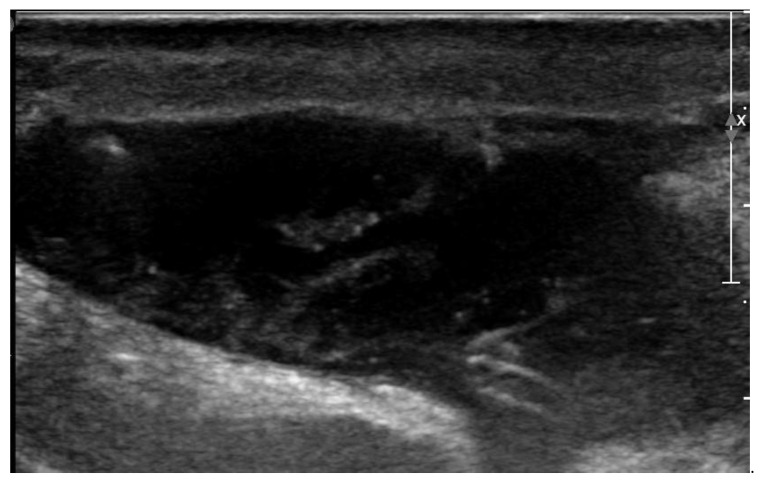
Two-dimensional grayscale ultrasonographic image of the swelling at the level of the surgical wound of the jugular venous incision shows a pocket of fluid accumulation.

After discharge, the postoperative period went uneventful, but on the fifth postoperative day the owner noticed lethargy, anorexia, and severe exercise intolerance, when the dog could hardly walk more than 25 m. The dog was presented to the emergency service of the authors’ institution at night. Physical examination revealed a lethargic dog with a respiratory rate of 30/min, a pulse rate of 60/min and a rectal temperature of 39.5 °C. Both wounds, on the right chest and over the right jugular vein region appeared normal, without redness, discharge or pain. However, a seroma formation was suspected around the cervical wound because this region was mildly swollen. With a blood test, a moderate leukocytosis was found (24.0 × 10^9^/liter, reference 4.5–14.6 × 10^9^/liter) due to neutrophilia (21.8 × 10^9^/liter, reference 2.9–11.0 × 10^9^/liter). Plasma creatinine (139 µmol/l, reference range 50–129 µmol/l) and lactate (2.89 mmol/l; reference 2.0–2.5 mmol/l) concentrations showed mild elevation and the plasma albumin concentration (21 g/l; references 26–37 g/l) was slightly decreased. Hematocrit and plasma biochemistry panel showed that blood urea nitrogen, total protein, potassium, sodium, chloride, calcium, glucose concentrations, and the pH were within the reference range. The dog was hospitalized. The next morning the cervical wound looked not only more swollen, but also it became red, warm, and painful. A bacterial infection of the subcutaneous part of the pacing lead was suspected and an ultrasonographic examination of the swollen cervical region was performed. Ultrasonography showed that the subcutaneous portion of the pacemaker lead was surrounded by a mild amount of anechoic fluid. At the region where the lead entered the right external jugular vein, a larger pocket of fluid (3.4 cm × 0.9 cm) was found, where the fluid was mainly anechoic with some echogenic threads (Figure 4). The subcutaneous fat around the fluid accumulation appeared diffusely hyperechoic. 

**Figure 5:**
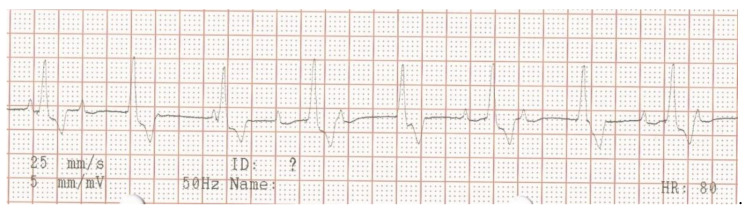
Surface electrocardiogram at the appearance of lethargy, 5 days after pacemaker implantation. Lead aVF shows paced ventricular QRS-complexes with 100% capture and atrio-ventricular dyssynchrony, compatible with an appropriately functioning pacemaker with ventricular single-chamber (VVIR) pacing mode.

Three blood samples for bacterial cultures were collected with one-hour intervals from three different peripheral veins after surgical preparation of the venipuncture sites (i.e., clipping the hair and disinfection of the skin with alcohol), to investigate the presence of a possible sepsis. The blood samples were submitted to the microbiology laboratory of the authors’ institution. Thereafter, parenteral antibiotic therapy with amoxicillin with clavulanic acid (12.5 mg/kg IV q8h) and enrofloxacin (5 mg/kg SC q24h) was started. The next day reddish brown discharge appeared from the cervical wound, which was sampled and submitted to bacterial culture. The dog’s general condition started to improve slowly, and the rectal temperature normalized. Electrocardiogram showed a third-degree AV-block and a good functioning pacemaker with only paced QRS-complexes and 100% capture (Figure 5). 

Echocardiography revealed no new findings, there were no signs of endocarditis or pulmonary thromboembolism. On the third day of hospitalization, the peripheral blood leukocyte counts normalized (12.7 × 10^9^/liter, reference 4.5–14.6 × 10^9^/liter). After four days of hospitalization, the dog was discharged with continuation of oral enrofloxacin and amoxicillin with clavulanic acid antibiotics. 

Culture of the three blood samples revealed no bacterial growth, but the culture of the reddish-brown fluid from the cervical wound revealed a moderate number of colonies of *Staphylococcus aureus*, which was sensitive to all tested antibiotics of the antibiogram. Oral amoxicillin with clavulanic acid (20 mg/kg q12h) and enrofloxacin (6 mg/kg q12h) were prescribed for three more weeks and a recheck examination was scheduled three weeks later.

**Figure 6:**
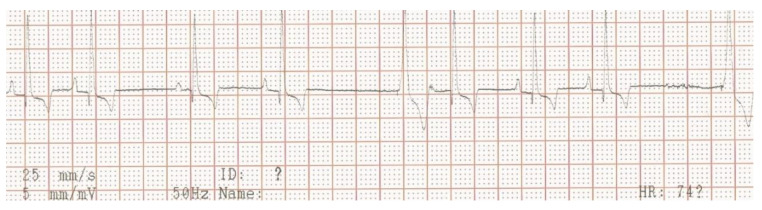
Surface electrocardiogram at the end of the first course of antibiotic therapy. Lead aVF shows sinus arrhythmia, where at the longer pauses a paced ventricular QRS-complex appears (VVIR pacing mode).

At the recheck examination the owner reported resolution of all previously noted clinical signs. Physical examination revealed no abnormal findings, except for the known cardiac murmur. The cervical region around the previous surgical wound showed no signs of inflammation anymore. Electrocardiography showed a respiratory sinus arrhythmia with a rate between 100–120/min as a basal rhythm, which was occasionally interrupted with short periods of 1–4 paced QRS-complexes (Figure 6). The antibiotic therapy was stopped after a total administration of 4 weeks.

**Figure 7:**
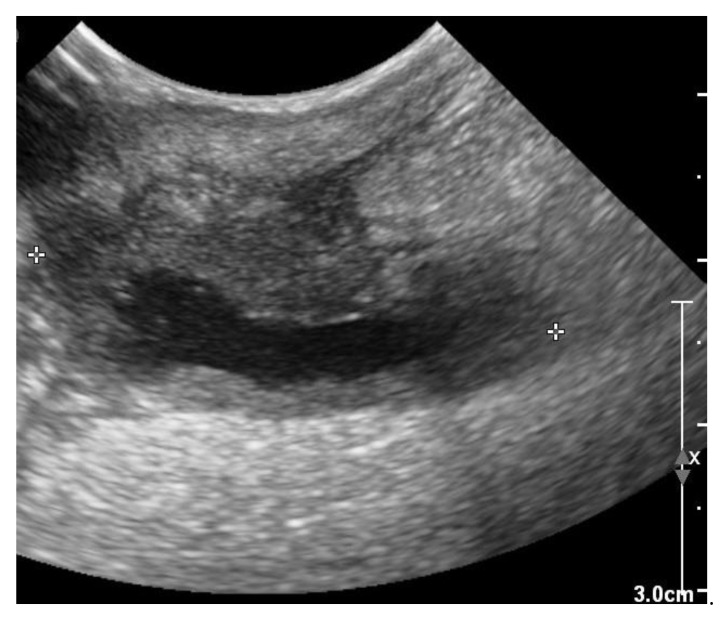
Two-dimensional grayscale ultrasonographic image of the swelling at the level of the previously healed surgical wound at the level of the jugular venous incision shows a pocket of fluid accumulation surrounded by abnormal soft tissue.

Three weeks after cessation of the oral antibiotic therapy, the dog became lethargic again and the owner noticed discharge from the previously healed cervical wound. The dog was presented again to the authors’ teaching hospital. Physical examination revealed a lethargic dog with a rectal temperature of 39.5 °C, and a warm and red lump of about 5 × 5 cm at the region of the previous surgical wound over the right jugular vein. In the skin overlying the lump, an opening of about 3 mm was visible, where purulent discharge was noticed. The dog was hospitalized again. Ultrasonographic examination of the lump was performed, which again identified a subcutaneous fluid pocket, but this time surrounded by a thick wall (Figure 7). 

Under ultrasound-guidance, a puncture with a fine needle was performed, and the fluid was sampled. Special caution was exercised to prevent puncturing the pacemaker lead by visualizing it with ultrasound. The gained fluid sample was submitted for bacteriologic culture and cytologic examination. Cytology revealed exudative inflammation without visible bacteria. Repeated echocardiography did not reveal any signs of endocarditis. Due to the suspicion of persistent bacterial infection of the pacing lead, explantation of the pacing lead and the pulse generator was considered, and oral amoxicillin with clavulanic acid (10.7 mg/kg q12h) therapy was re-started. Explantation of the pacemaker seemed to be a safe option in this dog because the high-degree AV-block spontaneously resolved, and sinus rhythm was present. To ensure that the dog would function sufficiently without a pacemaker, the pulse generator was first switched off by programming it to OOO mode. Electrocardiography (ECG) revealed respiratory sinus arrhythmia with short periods of second- and third-degree AV-block with the lowest ventricular rate of 50 beats/min. The dog was discharged from the hospital with a Holter ECG for the weekend with the pacemaker in OOO mode. After the dog experienced a syncopal episode at home, it was brought back to the authors’ institution, and the pacemaker was switched on again and programmed in VVIR mode. 

**Figure 8:**
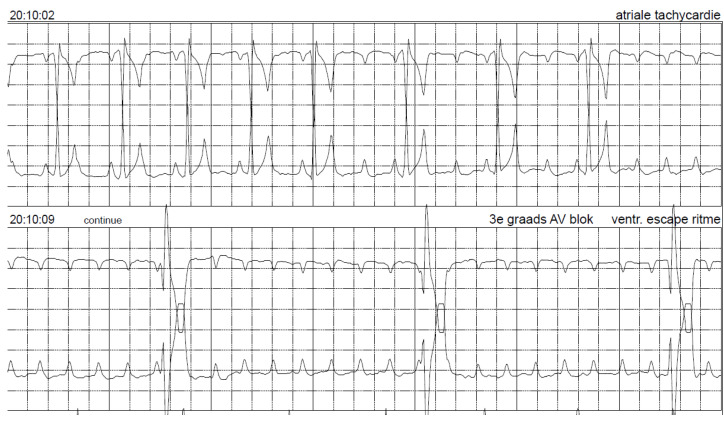
Holter electrocardiogram while the pacemaker was switched off (OOO mode) shows sinus rhythm, which turns into second- and then third-degree atrio-ventricular block. Paper speed 25 mm/s.

The Holter ECG registration showed sinus arrhythmia as basic rhythm and longer periods of severe bradycardia due to intermittent second- and third-degree AV-block (Figure 8).

The bacterial culture of the fluid gained under ultrasound-guidance from the subcutaneous pocket revealed a low number of colonies of *Staphylococcus aureus*, which was sensitive to all tested antibiotics. The amoxicillin with clavulanic acid was stopped after a week and oral clindamycin (11.3 mg/kg q8h) was started because (1) the tissue penetration of this antibiotic agent is known to be superior compared to amoxicillin, (2) based on the antibiogram it was expected to be effective too, (3) this antibiotic is approved for treating skin abscesses in dogs for a maximum of 28 days with a recommended dose range of 5.5–33 mg/kg q12h by the American Food and Drug Administration, and (4) this antibiotic is considered less essential for human use than amoxicillin according to the World Health Organization from the antibiotic-resistance point of view [20,21,22].

**Figure 9:**
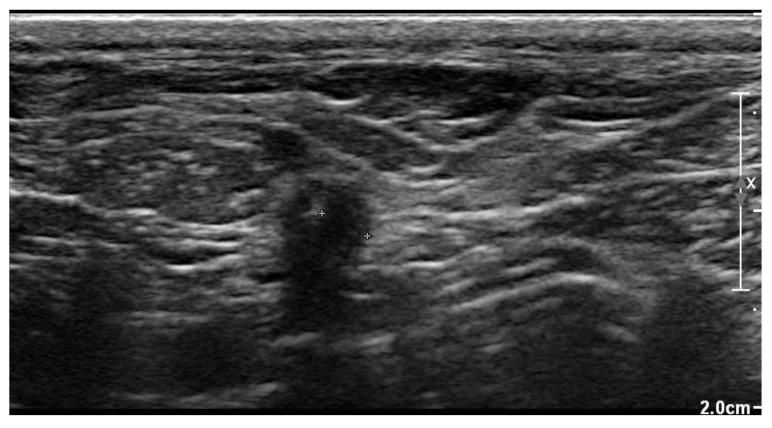
Two-dimensional grayscale ultrasonographic image at the site of the previous swelling at the level of the jugular venous access shows a hypoechoic tissue of about 2.5 mm adjacent to the pacing lead, which is displayed in cross section.

The recheck examination 2 weeks later revealed no abnormalities with inspection and palpation of the region where previously the wound was located over the right jugular vein. Ultrasonography showed no subcutaneous fluid accumulation around the pacing lead, but there was a small region of hypoechoic tissue visible (Figure 9).

Clindamycin administration was continued until the next recheck examination, again 2 weeks later. At this time the dog was still doing clinically good and ultrasonographic changes around the cervical part of the pacing lead showed no fluid accumulation; however, the hypoechoic tissue was still present. Under ultrasound guidance a fine-needle aspiration biopsy was taken from this hypoechoic tissue. Again, specific caution was exercised to avoid puncturing the pacing lead. Cytologic examination revealed no inflammatory cells. Based on this finding, clindamycin therapy was stopped after a total of 4 weeks of administration. Recheck examination one month later was scheduled. During the recheck, the owner reported no apparent problems. Clinical examination revealed no abnormalities, including inspection and palpation of the cervical region where previously the infected wound was located. Ultrasonographic evaluation of the pacing lead at the cervical region showed no abnormalities either. Electrocardiography revealed a good functioning pacemaker in VVIR mode and a continuously present third-degree AV-block as underlying rhythm.

Recheck examination 6 months later was conducted. The owner reported that the dog was still doing well. Physical examination revealed no abnormalities. 

Ten months after the last recheck, at the age of 14.5 years, the dog was euthanized by the referring veterinarian because of behavior problems most likely arising from dementia, and chronic progressive paresis posterior. At the time of euthanasia, no bradycardia was present, no syncopal episodes were reported, and no signs of possible infection were present at the sites of the previous surgical wounds.

## 3. Discussion

Despite routine administration of prophylactic antibiotics during pacemaker implantation procedures, typically a cephalosporin given intravenously, device infection still takes place in humans and in dogs. The prevalence of pacemaker infection in humans is reported to be around 1% [14,15,16,17,18,19], whereas in dogs 5–10% [1,8,9,12]. Based on a large scale double-blind, placebo-controlled randomized clinical trial in humans, prophylactic antibiotic use is established in preventing pacemaker infection [17]; however, such a study has not been performed in dogs yet. Multivariate analysis in human studies on pacemaker infection revealed hematoma formation and lack of antibiotic prophylaxis as two independent predisposing variables for device-infection [14,15,16,17,18,19]. In both veterinary and human medicine, after hour emergency surgeries were identified as an additional contributing factor to pacemaker infection [12,14,15,16,17,18,19]. In humans, there are various protocols regarding the length of antibiotic prophylaxis used, ranging from a single intraoperative injection given before skin incision, through 1-day to 3-day antibiotic courses [14,15,16,17,18,19,23,24]. As scientific evidence in veterinary medicine is lacking, expert opinion and extrapolation of human recommendations are applied to dogs regarding the use of prophylactic antibiotics at the perioperative period of implantation of permanent pacemakers. One of the most widespread used veterinary textbooks on this topic does not even recommend the routine administration of prophylactic antibiotics in dogs that undergo pacemaker implantation [1]. 

In the present case report empirical antibiotic choice of a potentiated beta lactam antibiotic combined with a fluoroquinolone did resolve the acute clinical signs of bacterial infection, but it failed to clear the infection on long-term. The port of bacterial entry was most likely the surgical site. Though human guidelines recommend surgical explanation of all parts of the pacemaker, i.e., lead and pulse generator, in case of bacterial infection of any part of a pacemaker, the dog in the present report had severe clinical signs attributable to bradycardia when the pacemaker was temporarily switched off for a couple of days. Therefore, surgical explanation of the pacemaker in this dog was considered unethical and hazardous. There are human cases reported, where successful antimicrobial medical management of a bacterial infection of the pacemaker is described without explanation of the device [25].

Ultrasonographic examination of the swelling around the pacing lead was an important diagnostic test to localize the subcutaneous fluid accumulation (i.e., abscess), and to guide the sampling fine-needle into the abscess without damaging the lead. Human guidelines recommend against culturing the fluid that spontaneously appears in the wound when the infectious agent is to be identified [14,15,16,17,18,19]. Repeated ultrasonographic examination of the subcutaneous tissue around the infected pacing lead and repeated ultrasound-guided fine-needle aspiration biopsies of the abnormally appearing subcutaneous tissue was performed in the present case to monitor for the persistence of inflammation, so that to facilitate decision making about the length of the antibiotic therapy.

Third-degree AV-block in dogs is typically a constantly present arrhythmia. However, in the present case, this was initially intermittent, and also paroxysmal third-degree AV-block caused the syncopal episodes. 

Clindamycin is a narrow-spectrum bacteriostatic lincosamide antibiotic agent, which is effective for infections caused by Gram-positive bacteria, such as *Staphylococcus*, a commonly encountered bacterium in surgical site infections [14,15,16,17,18,19,22,23,24]. *Staphylococci* isolated from the canine skin are shown to have a high risk for biofilm formation [26]. Biofilm formation on implants, including pacing leads and pulse generators, can make antimicrobial therapy of device-infection challenging. Clindamycin is classified as a highly important drug according to the World Health Organization (WHO) [21]. On the other hand, amoxicillin with clavulanic acid, which is more often used in dogs as first choice antibiotics, is categorized as critically important antibiotic by the WHO [21]. To fight against the increasing global threat of antibiotic resistance, it is of major importance to choose the most appropriate antibiotic agent and administer it at the right time and as short as possible, preferably based on the results of an antibiogram [22,23,24,25]. Potentiated beta lactam antibiotics, but particularly fluoroquinolones should be reserved for infections where no effective alternative antibiotics are available based on the antibiogram. Another reason to switch to another antibiotic in the present case was that repeated courses of the same antibiotic agent may select resistant bacterial strains, especially in case of an abscess.

The approach described in the present case report to manage device-infection is new, and it is in several aspects not in line with the recommended human and veterinary guidelines. According to our knowledge, this is the first case that successful medical management of a pacing lead infection is reported with the utilization of ultrasound-guided puncture of the infected tissue, without the need of explantation of the device after a failed course of empirical antibiotics. Repeated recheck examinations and ultrasound-guided punctures of the surgical site infection helped to establish the necessary length of antibiotic therapy. We also conclude that clindamycin resulted in the present case a permanent eradication of bacterial surgical site infection, unlike amoxicillin with clavulanic acid combined with enrofloxacin. As *Staphylococcus* is the most likely agent involved in surgical site infections, clindamycin should be the first choice in case of pacemaker infection, as the penetration of this molecule to inflamed and scar tissues, particularly abscesses, and to white blood cells is superior [20,21]. In addition, the hazard of selecting resistant bacterial strains against an essential antibiotic is lower, compared to the use of the broad-spectrum potentiated amoxicillin or a fluoroquinolone [20,21]. 

Extrapolating the current human guidelines to dogs in preventing surgical site infection after permanent pacemaker implantation, a single dose of intravenously administered cephalosporin given before the surgical incision is made, seems a reasonable approach [14,15,16,23,24]. Longer than 24 hours of postoperative administration of prophylactic antibiotics after pacemaker implantation seems unnecessary based research in humans [24].

## 4. Conclusions

The present case report points out how a carefully chosen narrow-spectrum antimicrobial agent, clindamycin, could successfully address a device infection, making surgical removal of the pacemaker unnecessary after a failed course with second- and third-choice antibiotics. Though *Staphylococcus* was the most likely bacterium responsible for this surgical site infection, ultrasound-guided fine-needle aspiration biopsy of the inflamed subcutaneous tissue provided valuable samples for bacteriologic and cytologic examinations, without damaging the pacing lead or worsening the local bacterial infection. During the antibiotic course, repeated cytologic examinations of the inflamed tissue, gained by ultrasound-guided punctures, helped to prevent an unnecessarily long antimicrobial therapy.

## Data Availability

The data presented in this study are available in the article.
